# Estimated collective effective dose to the population from nuclear medicine diagnostic procedures in Croatia: A comparison of 2010 and 2015

**DOI:** 10.1371/journal.pone.0180057

**Published:** 2017-06-29

**Authors:** Ivana Kralik, Mario Štefanić, Hrvoje Brkić, Gordan Šarić, Stanko Težak, Svjetlana Grbac Ivanković, Neva Griotto, Damir Štimac, Otmar Rubin, Tamer Salha, Zrinka Ivanišević, Slaven Jurković, Dario Faj

**Affiliations:** 1 State Office for Radiological and Nuclear Safety, Zagreb, Croatia; 2 Faculty of Medicine, Josip Juraj Strossmayer University of Osijek, Osijek, Croatia; 3 University Hospital Centre Osijek, Osijek, Croatia; 4 University Hospital Centre Zagreb, Zahreb, Croatia; 5 University Hospital Centre Rijeka, Rijeka, Croatia; 6 Faculty of Medicine, University of Rijeka, Rijeka, Croatia; ENEA Centro Ricerche Casaccia, ITALY

## Abstract

**Objective:**

This study presents national surveys of patient exposure from nuclear medicine (NM) diagnostic procedures in 2010 and 2015 in the Republic of Croatia.

**Methods:**

The survey was performed according to the European Commission Dose DataMed (DDM) project methodology. 28 most frequent NM diagnostic procedures were identified. Data about frequencies of procedures and average administered activities of radioisotopes used in those procedures were collected. Average administered activities were converted to effective doses according to the dose conversion coefficients. Then the collective effective dose to the population and an effective dose per capita were calculated based on the number of the most frequent NM diagnostic procedures and the average effective dose per procedure.

**Results:**

In 2010, 41200 NM diagnostic procedures led to 146.7 manSv collective effective dose to the population and in 2015, 42000 NM diagnostic procedures led to 146.8 manSv collective effective dose to the population. The frequencies of NM diagnostic procedures were 9.7 and 9.8 annually per 1000 population with 34.1 μSv and 34.2 μSv effective dose per capita for 2010 and 2015, respectively. The main contributors to the annual collective dose from NM in Croatia are examinations of the bone, heart, thyroid and PET/CT tumour diagnostic. Average administered activities have not changed considerably from 2010 to 2015. Nevertheless, within the frequency of some of the procedures, significant changes were found in five-year period.

**Conclusions:**

Frequencies, average administered activities and collective effective dose to the population from NM diagnostic procedures in Croatia are comparable to the values reported by other European surveys. Changes were found between 2010 and 2015 and we intend to perform this study periodically to identify possible trends, but also to raise awareness about the potential dose optimization.

## Introduction

The largest contribution to the man-made radiation exposure of population comes from the medical exposure and it grows continuously [1–3]. Though the major part of medical exposure comes from diagnostic X-rays, it has been shown that contribution of nuclear medicine (NM) diagnostic procedures in most developed countries is between 4% and 14% [4–6].

The study on medical exposure in the European population gave the guidance on the implementation of the Article 12, Medical Exposure Directive [7, 8]. In the follow-up project, Dose DataMed2 (DDM2) [6], Croatia was selected to participate as a test country because of its less experience in population dose estimations. In 2010, a workgroup of radiologists, nuclear medicine specialists, medical physicists and a statistician was formed and worked together with national radiation safety regulatory body (State Office for Radiological and Nuclear Safety, SORNS). Implementation of the European guidance [7] was tested. Therefore, in 2010 a survey about the population exposure from diagnostic radiology and NM examinations was performed for the first time in Croatia. The same survey was repeated in 2015.

This study summarizes and compares the results of surveys of patient exposure from NM diagnostic procedures in 2010 and 2015 in the Republic of Croatia.

## Materials and methods

Population dose from medical exposure is given as annual collective effective dose and the annual average per caput effective dose from radiodiagnostic procedures [7]. Usefulness of estimation of population dose lies in the fact that it allows radiation protection and healthcare authorities to observe trends in annual collective dose from medical exposure and contribution from each imaging modality and types of diagnostic procedures to the total collective dose from all radiodiagnostic procedures, to determine the relationship between the frequencies of different radiodiagnostic procedures, the radiation doses given to patients and their contribution to the collective dose thus indicating possible need for optimisation of the protection of most highly exposed patients [7]. Shortcomings of this concept are related mainly to the resources required, availability of information on the annual numbers of all important types of procedures, choice and availability of suitable conversion coefficients and need for careful evaluation of uncertainties. In cases when population dose is used for comparison of contribution from radiodiagnostic procedures with those from other natural and man-made sources, limitations of collective effective dose related to age/sex distribution should be considered [7]. In addition, although population dose from medical exposure is derived from averaged effective dose to the exposed group, it should not be used for assessing radiation risks to populations of patients by simple application of the nominal probability coefficients for radiation-induced cancer which have been derived for a general population. This is due to non-uniform dose distribution throughout the body which may lead to variation with age at exposure and sex of radiation risk for different organs different than in case of uniform whole body exposure [7]. Instead, having information on the age and sex distribution of patients undergoing the types of x-ray examination making major contributions to the total collective dose, for relating the collective doses to the collective detriment age, sex- and organ-specific radiation risk models should be used [7].

According to the census of 2011, Croatia has the population of 4290000 citizens with 13760$ of GDP per person (International monetary fund in 2010). With over 2.5 physicians per 1000 population Croatia belongs to the health level one countries according to the UNSCEAR classification.

There are 13 NM departments in Croatia; nine of them are large and four perform only a limited number of examinations or non-imaging procedures. Eleven are public and two private institutions.

The collective effective dose to the Croatian population from the NM exposure was calculated based on the number of medical procedures and the effective dose per procedure. According to the DDM methodology [7, 8], the number of medical procedures were collected for the 28 most frequently used procedures in NM. Out of these 28 procedures, three NM diagnostic procedures were not performed in Croatia (Myocardial perfusion using PET and dopamine transporter imaging (parkinsonism) using ^123^I, ß-CIT). On the other hand, three other NM examinations, considered to be relevant in Croatia, were added, leading to final 28 NM examinations included in the study.

In 2010 no distinction between different kinds of PET examinations was considered since the data from private owner were given only as a total number of procedures, and in 2015 the survey distinguishes between PET and PET/CT examinations. However, exposure from the CT part of the PET/CT examinations was not included, although it contributes around a half of the total PET/CT dose to the patient [9].

At first, the number of medical procedures was obtained from the Croatian Health Insurance Fund (CHIF). The CHIF database covers more than 99% of population and is used primarily for reimbursement purposes. To check the usability of CHIF data we also conducted direct survey in 7 out of 9 large NM departments (private institutions were not included = > no direct survey of PET/CT examinations number) in 2010, and in 8 out of 9 large NM departments with all four small NM departments in 2015. Then surveyed data were compared to CHIF data. Most of the results complied within 10%, but e.g. bone scintigraphy data showed that CHIF results overestimated the number of examinations done by factor 3. The reason for this is that in CHIF counts not only the administered activity, but also the number of procedures performed per single application of a radiopharmaceutical. In addition, an error was likely introduced by variable coding practices and unverified claims coded by the NM centers themselves. This makes CHIF data ambiguous for the purpose of this study. Nevertheless, since similar errors and differences between surveyed and CHIF data was found in all surveyed NM departments it was reasonably to assume that the same differences would be found in the rest of the NM departments. For this reasons we decided to use the CHIF data only to extrapolate the number of NM procedures from surveyed NM departments to all NM departments. In 2010, extrapolation coefficient for total number of NM examinations was 1.19 and in 2015 it was 1.08. It complied with our subjective estimation according to the size of surveyed and non-surveyed departments. The same extrapolation method was used to estimate some missing monthly collected data for other hospitals.

Average administered activities for the most frequent NM examinations were surveyed in the same departments and converted to the effective doses according to the ICRP dose conversion coefficients [6, 10–12] or product specifications given by manufacturers.

The collective effective dose was then calculated for each NM procedure as a product of the frequency and the average effective dose per procedure.

### Uncertainties of the results

The collected data are representative sample of NM diagnostic procedures performed in Croatia. Analyzed procedures were estimated to contribute over 80% to the total collective effective dose from NM examinations in Croatia. The uncertainty estimation was done according to the DDM2 methodology [6–8]. The recognized sources of uncertainties were the uncertainties in the number of procedures, of typical average administered activities and mean uncertainty of conversion factors [6]. Since small part of NM departments in Croatia did not reported the data and extrapolation was done according to the CHIF data, we conservatively estimated uncertainty of extrapolated data to be 50%. For all other NM departments the frequency data were collected and the uncertainty of data was estimated to be 5%. The mean uncertainty of average administered activities and of conversion factors was used as recommended in the DDM2 report [6] to be 10% and 20%, respectively. The total uncertainty estimation took into account relative contributions and conservative assumptions of uncertainties for each department. Following these assumptions the total uncertainties on the collective effective dose was estimated to be less than 10% (at 95% confidence level) and less than 7% (at 95% confidence level) for 2010 and 2015 surveys, respectively.

## Results

Collected frequencies and the mean administered activity for the most used NM procedures in Croatia together with effective dose conversion coefficients are presented in the Table 1. In the columns, list of types of examinations with radionuclide and chemical form of radiopharmaceutical used is given. Then the number of examinations performed yearly, average administered activity per examination, dose conversion factors, the effective dose per examination and contribution of each examination to the collective effective dose from NM diagnostic procedures are given for both surveyed years.

**Table 1:** Data of NM diagnostic procedures in Croatia, surveyed in 2010 and 2015.

	Examination	Radionuclide	Chemical form	Number of exam-inations (adults) 2010	Number of exam-inations (adults) 2015	Mean activity per examination 2010 (MBq)	Mean activity per examination 2015 (MBq)	Conversion factor (mSv/MBq)	Effective dose per exam-ination (mSv) 2010	Effective dose per exam-ination (mSv) 2015	Collective effective dose (manSv) 2010	Collective effective dose (manSv) 2015
1.	Bone imaging	^99m^Tc	Phosphates and phosphonates	10928	11404	592 (480–740)	633 (480–740)	5.70E-03^11^	3,37	3,61	36,88	41,15
2.	Myocardial perfusion	^201^Tl	Chloride	396		75 (40–111)		1.40E-01^12^	10,5		4,16	0
3.	Myocardial perfusion, rest	^99m^Tc	Tetrofosmin	962	353	572 (555–740)	578 (555–600)	6.90E-03^12^	3,95	3,99	3,8	1,41
4.	Myocardial perfusion, exercise	^99m^Tc	Tetrofosmin	995	572	560 (555–600)	577 (555–600)	6.90E-03^12^	3,86	3,98	3,84	2,28
5.	Myocardial perfusion, rest	^99m^Tc	MIBI	1706	2797	608 (555–850)	564 (400–800)	9.00E-03^11^	5,47	5,08	9,33	14,2
6.	Myocardial perfusion, exercise	^99m^Tc	MIBI	930	2282	635 (555–850)	555 (400–800)	7.90E-03^11^	5,02	4,38	4,67	10
7.	Tumor imaging (PET)	^18^F	FDG		3761		232 (200–240)	1.90E-02^12^		4,41	0	16,58
8.	Tumor imaging (PET) + Diagnostic CT	^18^F	FDG		5011		226 (200–240)	1.90E-02^12^		4,29	0	21,52
9.	Thyroid metastases (after ablation, uptake 0%)	^131^I	Iodide	830	890	185 (185–185)	167 (111–185)	6.10E-02^11^	11,3	10,2	9,37	9,06
10.	Thyroid imaging (oral administation, no blocking)	^99m^Tc	Pertechnetate	9563	7012	117 (75–185)	98 (75–185)	1.30E-02^11^	1,52	1,27	14,55	8,93
11.	MUGA, cardiac blood pool, cardiac blood flow (equilibrium)	^99m^Tc	DTPA	160		740 (740–740)		4.90E-03^11^	3,63		0,58	0
12.	MUGA, cardiac blood pool, cardiac blood flow (equilibrium)	^99m^Tc	Tc-labelled eryhrocytes	7		801 (555–925)		7.00E-03^11^	5,61		0,04	0
13.	Dopamine transporter imaging (parkinsonism)	^123^I	Ioflupane (DaTscan)	148	63	122 (111–130)	113 (111–150)	2.40E-02^12^	2,93	2,71	0,43	0,17
14.	Lung perfusion	^99m^Tc	MAA	1578	1121	150 (111–185)	154 (111–185)	1.10E-02^11^	1,65	1,69	2,6	1,9
15.	Neuroendocrine tumors/somatostatin receptor imaging	^111^In	Pentetreotide (OctreoScan)	101		240 (185–555)		5.40E-02^12^	13		1,31	
16.	Renal imaging	^99m^Tc	DMSA	1092	435	105 (74–185)	101 (74–148)	8.80E-03^11^	0,92	0,89	1,01	0,39
17.	Renal imaging	^99m^Tc	MAG 3	1879	2151	120 (74–145)	140 (80–185)	7.00E-03^11^	0,84	0,98	1,58	2,11
18.	Renal imaging	^99m^Tc	DTPA	1192	707	142 (111–370)	181 (103–370)	4.90E-03^11^	0,7	0,89	0,83	0,63
19.	Parathyroid imaging	^99m^Tc	MIBI	695	787	536 (370–740)	510 (300–740)	9.00E-03^11^	4,82	4,59	3,34	3,61
20.	Cerebral blood flow	^99m^Tc	Exametazime (HMPAO, Ceretec)	65	307	740 (740–740)	855 (600–1110)	9.30E-03^11^	6,88	7,95	0,45	2,44
21.	Cerebral blood flow	^99m^Tc	ECD (Neurolite)	252		850 (850)		2.20E-03^12^	1,87		0,47	0
22.	Infection/inflamation imaging	^67^Ga	Gallium citrate	338	35	72 (55–84)	77 (74–80)	1.00E-01^11^	7,7	7,7	2,43	0,27
23.	Infection/inflamation imaging	^99m^Tc	Tc-labelled white blood cells (leucocytes)	144	260	740 (740)	555 (555)	1.10E-02^11^	8,14	6,11	1,17	1,58
24.	Infection/inflamation imaging	^99m^Tc	Monoclonal antibody (LeucoScan)	113		555 (555–555)		8.00E-03^12^	4,44		0,51	0
25.	Lymphoscintigraphy (sentinel & other)	^99m^Tc	nanocollid	227	927	23 (11–25)	26 (4–30)	1.70E-02^4^	0,34	0,33	0,03	0,12
26.	Angiocardiography—SHUNT	^99m^Tc	Pertechnetate	83	227	796 (555–925)	771 (500–1110)	1.30E-02^12^	11,1	10,8	0,93	2,45
27.	Liver hemangioma	^99m^Tc	Tc-labelled erytrocites	280	878	631 (555–740)	570 (555–800)	7.00E-03^12^	4,42	3,99	1,23	3,5
28.	PET—CT all	FDG		6559		330 (330)		1.90E-02^12^	6,27		41,13	
** **	**Total**	** **	** **	**41223**	**41980**	** **	** **	** **	** **	** **	**146,98**	**146,82**

In years 2010 and 2015, a total of 41200 and 42000 NM examinations were performed respectively, resulting in 146.7 manSv and 146.8 manSv collective effective doses. This corresponds to 9.7 and 9.8 examinations per 1000 population annually with 34.1 μSv and 34.2 μSv effective doses per capita in 2010 and 2015, respectively. Based on an estimated number of diagnostic radiology examinations for the same periods in Croatia (unpublished SORNS study), NM examinations contributed approximately 1.5% to the total frequency of all diagnostic medical procedures (excluding dental). NM contributions to the collective effective dose from diagnostic medical examinations (excluding dental) were approximately 6% in both years. The overall number of examinations and total collective effective dose difference between 2010 and 2015 were within the data uncertainty, but significant changes within some NM diagnostic procedures from 2010 to 2015 exist.

**Fig 1:**
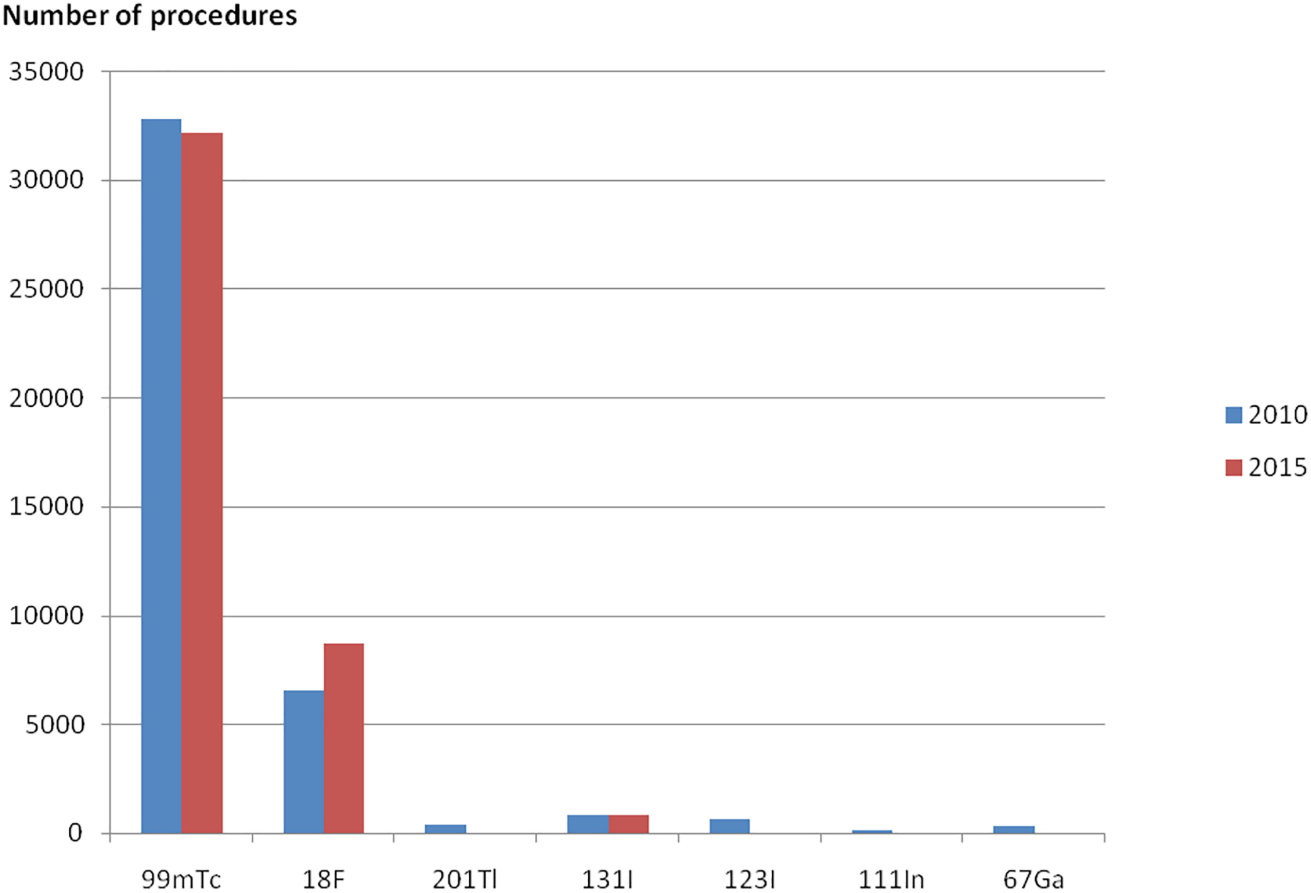
Number of NM diagnostic procedures according to the isotope used. Blue bars are representing data of 2010 and red bars data of 2015.

Croatian NM departments used mostly ^99m^Tc, ^18^F, ^131^I, ^123^I, ^201^Tl, ^111^In and ^67^Ga radioisotopes in 2010 and 2015. Fig 1 shows that ^99m^Tc is the most used in NM diagnostic procedures in Croatia contributing with more than 75% in total number of procedures in 2010 as well as in 2015. The ^18^F contributed over 15% to the total number of procedures in 2010 and contribution rose to over 20% in 2015. In the mean time, the radioisotope ^201^Tl was practically abandoned in surveyed departments; the turnover of ^111^In sharply decreased and the number of procedures using ^67^Ga was significantly reduced in 2015.

**Fig 2:**
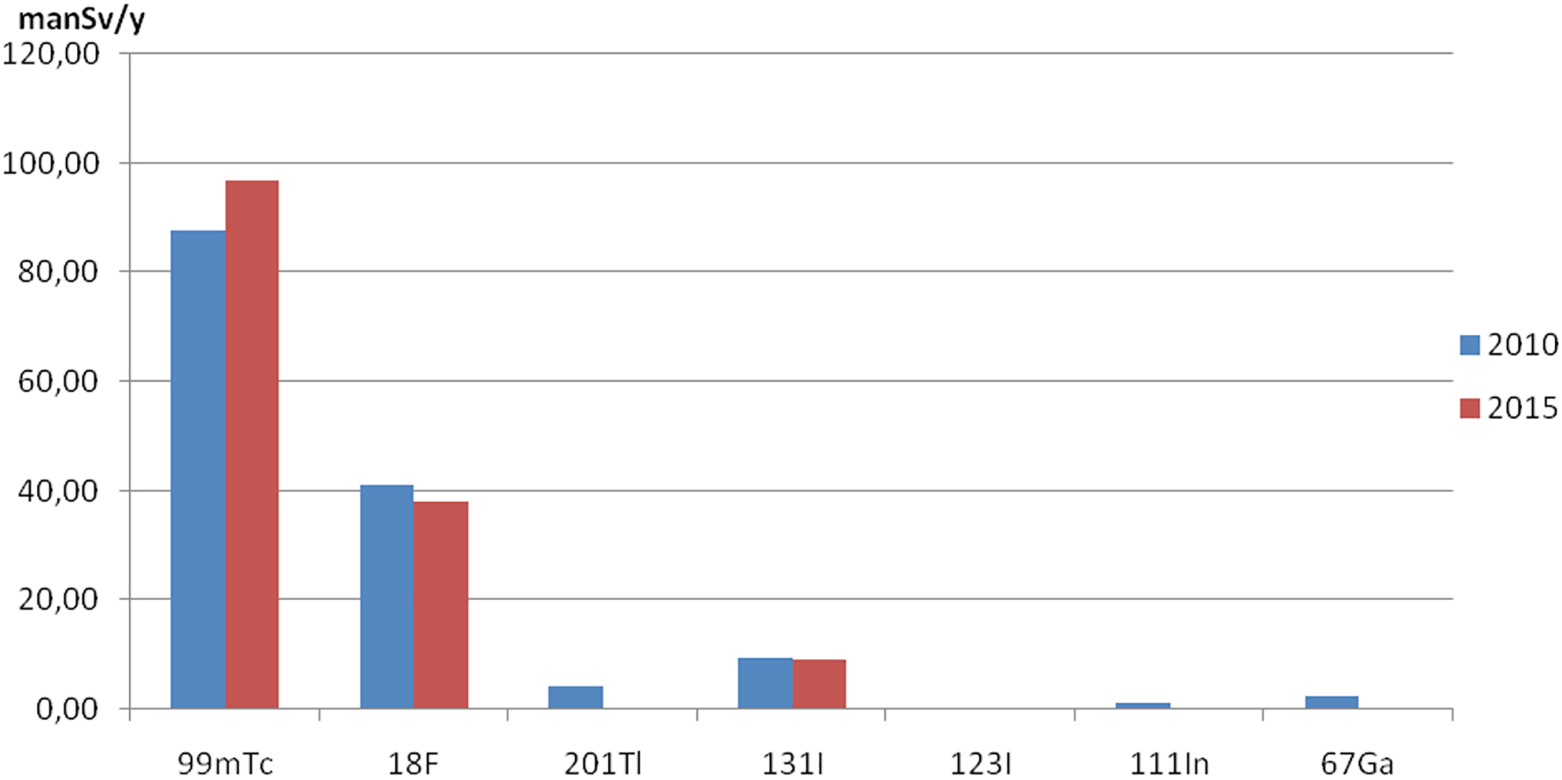
Collective effective dose from NM diagnostic procedures according to the isotope used. Blue bars are representing data of 2010 and red bars data of 2015.

Fig 2 shows contributions to the total collective effective dose from NM procedures according to the isotope used. It could be seen that procedures with higher effective dose per procedure contributes more here. ^99m^Tc contributes 60% and 65% to the total collective effective dose in 2010 and 2015, respectively. On the other hand, ^18^F contribution is more than 25% in both years.

**Fig 3:**
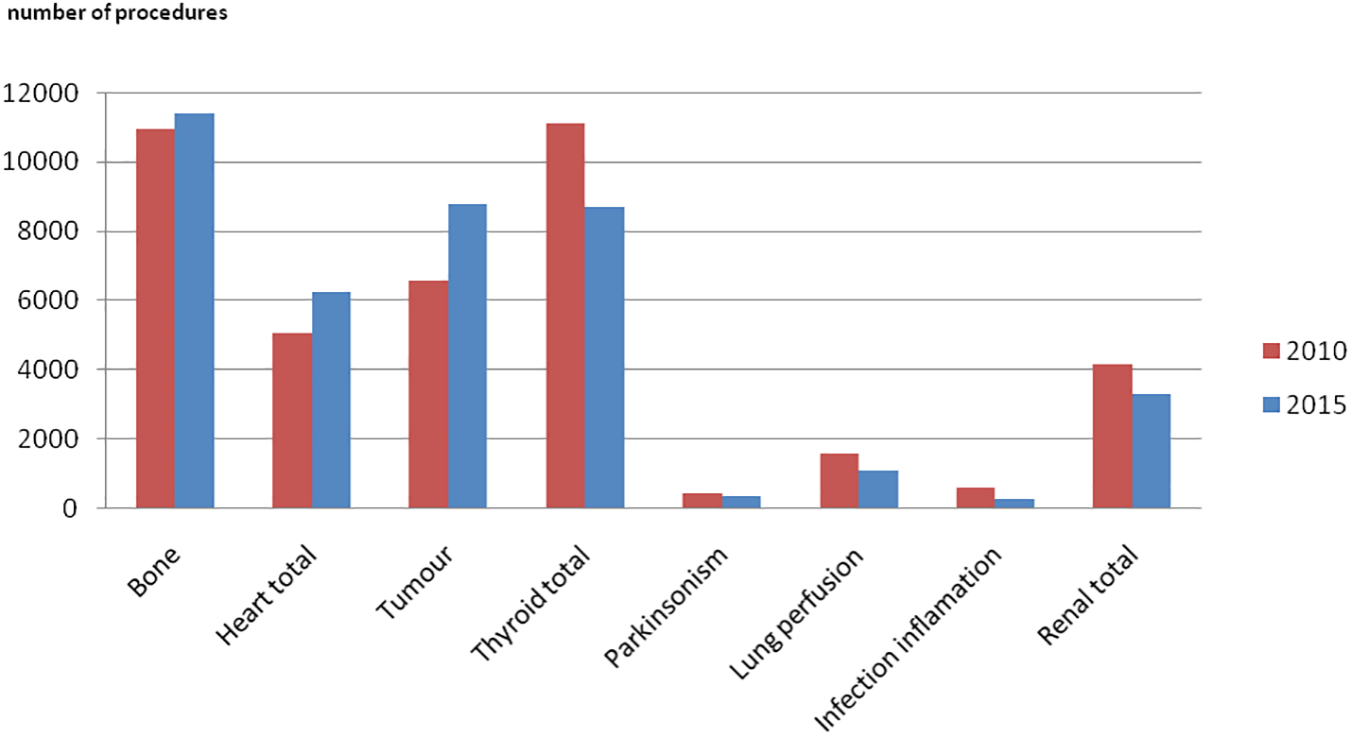
Number of NM diagnostic procedures grouped according to the organ, target or closely similar objectives. Red bars are representing data of 2010 and blue bars data of 2015.

**Fig 4:**
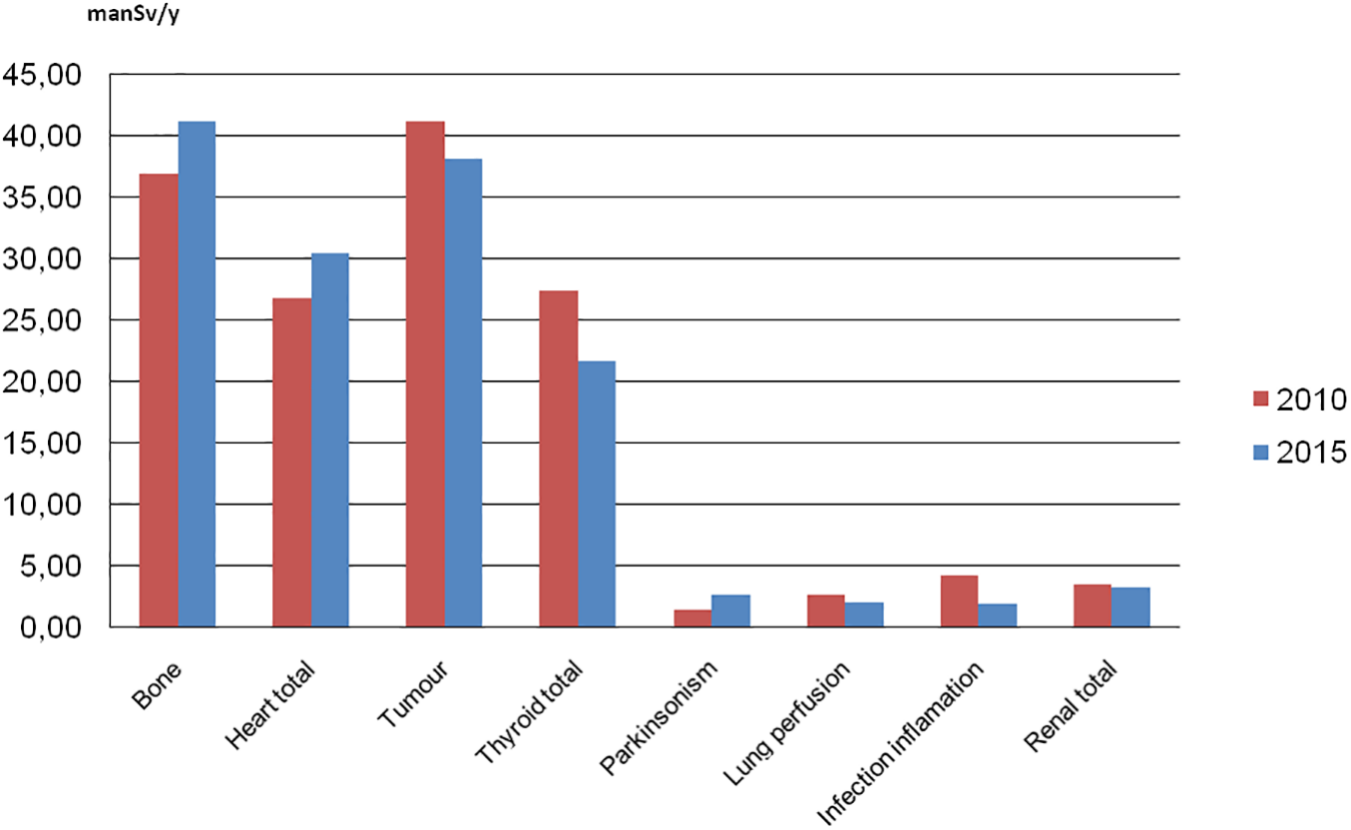
Collective effective dose from NM diagnostic procedures grouped according to the organ, target or closely similar objectives. Red bars are representing data of 2010 and blue bars data of 2015.

According to the data, we determined contributions to the total number of NM diagnostic procedures and to the total collective effective dose from different examinations group, grouped together according to the organ, target or closely similar objectives as proposed before [6]. Procedures were grouped as bone, heart, thyroid, tumor imaging, infection/inflammation, lung perfusion, parkinsonism and renal total. The distributions are given in the Figs 3 and 4 for both surveyed years.

Bone scans, heart, thyroid examinations and tumor imaging contribute over 80% to the total annual number of NM diagnostic procedures and over 90% to the total collective effective dose in both surveyed years.

Comparison of average administered activities of radioisotopes between the two surveyed years shows that most of procedures use the same values. Nevertheless, for some examinations concerned, such as somatostatin receptor imaging, cerebral flow imaging and infection/inflammation imaging, we observed the changing patterns of radiopharmaceutical use: ^**111**^In-pentetreotide, ^**99m**^Tc-ethyl cysteinate dimer (ECD) and ^**99m**^Tc-antigranulocyte monoclonal antibodies were completely supplanted by ^**99m**^Tc-EDDA/HYNIC-Octreotate, ^**99m**^Tc-HMPAO and ^99m^Tc-HMPAO white blood cells, respectively. In addition, a shift from tetrofosmin and diethylene-triamine-pentaacetate (DTPA) towards methoxy-iso-butyl-isonitril (MIBI) and mercaptoacetyltriglycine (MAG3) was seen in myocardial perfusion imaging (2-day ^**99m**^Tc protocol) and dynamic renal scintigraphy, respectively. These differences aside, an increased demand for myocardial perfusion imaging and oncological PET/CT was prominent, but numerically largely offset by decreased usage of ^**99m**^Tc thyroid imaging, dopamine transporter imaging, lung perfusion scintigraphy, and renal dimercaptosuccinic acid (DMSA) scan in 2015. The total number of bone, dynamic renal, parathyroid, brain perfusion and infection/inflammation scans did not vary considerably over the same period.

Lastly, an important difference was found in tumor imaging where average administered activity of ^18^F decreased from 330 MBq to 220 MBq decreasing effective dose per procedure 30%. This affects all data represented in Figs 1, 2, 3 and 4 since contribution to number of procedures increased from 2010 to 2015, but contribution to the collective effective dose decreased.

## Discussion

This study presents first data of collective effective dose in NM diagnostic procedures according to the DDM2 methodology in Croatia. We estimated NM diagnostic procedures contribution to the total collective effective dose, but also compared trends in frequencies and average administered doses to patients in 2010 and 2015.

The total annual number of NM diagnostic procedures per 1000 inhabitants was found to be fewer than 10 in both years. This is in the lower range of previously published European data (from 8 to 56 NM procedures per 1000 inhabitants) [6, 8]. Also, the reported frequencies of NM diagnostic procedures in both 2010 and 2015 are in the range of published studies [4–6, 8, 9, 13, 14], except for a significantly higher frequency of thyroid examinations. This might partially reflect a burden of longstanding iodine deficiency and consequently increased population prevalence of thyroid nodularity [15]. Indeed, Croatia has been considered iodine sufficient since year 2003; therefore, only a negligible part of the population has spent entire life consuming adequately iodized salt. Nevertheless, a decline in total number of thyroid scans was observed over a 5 yr span, most likely reflecting a widespread use of thyroid ultrasound in Croatia. An additional effort is mandatory to attain compliance with the national guidelines on rational diagnosis of thyroid disorders [16].

The ranges and the means of administered activities and average typical effective dose per diagnostic NM procedure (mSv) of this study, in both surveyed years do not show any significant change and they are in the range of published values as well [4–6, 8, 9, 13, 14]. Ratios between maximal and minimal average administered activity were found to be less than three and similar in both surveyed years. Such high ratios have already been shown [6, 8], but it could be caused by poor performance of imaging instrumentation, different physician experience, variations in system geometries and collimation, or by outdated equipment and should be carefully investigated. In some instances, such as FDG, a reduction of administered activity with the completion of the learning curve was noticeable in 2015 survey. The average annual effective dose per capita from NM diagnostic procedures was 34 μSv what is in the lower range of published European data (30 μSv to 200 μSv [6, 8]).

Comparison with an unpublished SORNS study of diagnostic radiology examinations for the same periods, we confirmed that diagnostic NM examinations make a small proportion in the total number of all diagnostic radiological and NM examinations together (1.5%) and larger contribution to the collective effective dose (6%). This puts NM diagnostic procedures in high dose procedures.

The main contributors to the annual collective dose from NM in Croatia are examinations of the bone, heart, thyroid and PET/CT tumour diagnostic. This complies well with TOP 7 procedures identified as being amongst the highest contributors to the total collective effective dose of NM procedures in all DDM2 countries [6]. The exception was myocardial perfusion using ^201^Tl (Chloride) that was not used in 2015 in Croatia at all.

Limitation of the study is that the data of CT doses (coming from PET/CT) were not included, though CT part of PET/CT examination delivers around a half of total PET/CT dose to the patient [6, 9]. This was omitted since we had no data of CT practices in NM, but in the future CT dose data should be included also.

In 2010, a study with the same purpose but different methodology was published [17]. The number of diagnostic NM procedures in that study was estimated according to the amount of imported radionuclides provided by the SORNS. Only the total number of NM diagnostic procedures could be estimated from this data. Also, the dose per typical examination was roughly estimated from imported activities of radionuclides without knowing chemical form and referral diagnosis. Despite these shortcomings both studies lead to similar results in total frequency of examinations (8–11 vs 9.7 examinations per 1000 members of population). On the other hand annual effective dose per capita estimated from the simplified methodology (6.8–7.9 μSv/caput) was about five times lower from the estimate of this study (34.1 μSv/caput). Yet another advantage of the DDM approach used in our study is the possibility of continuously following the typical activities, radiopharmaceuticals in use and frequencies for each examination. This information can be useful in evaluation of practice.

The comparison of 2010 and 2015 data showed no significant overall frequency and dose change considering the uncertainty of methodology. Nevertheless, significant changes were found in the frequencies of number examinations in individual major diagnostic categories (Table 1, Figs 1–4). A decline in the number of ^67^Ga, ^201^Tl, ^99m^Tc tetrofosmin, multigated acquisition (MUGA) cardiac blood pool, lung perfusion, dopamine transporter imaging, renal DMSA and monoclonal antibody/besilesomab-based infection studies has been observed since 2010. The factors most likely behind the observed trends could be identified as:

1. a rapid proliferation of PET/CT centers (^67^Ga), X-ray multidetector computed tomographic pulmonary angiography (lung perfusion scintigraphy), cardiac magnetic resonance and real-time three-dimensional echocardiography (MUGA cardiac blood pool) across the country,
2. criticism regarding the high frequency of non-diagnostic scans (planar lung perfusion scintigraphy; V/Q scanning using SPECT and SPECT/CT is seldom used for investigation of pulmonary embolism in Croatia),
3. limited image quality, complex pharmacokinetics or instrumentation, unfavorable radiation dosimetry (^201^Tl, ^67^Ga, ^111^In),
4. superior diagnostic information (^99m^Tc MAG3 vs DTPA, renal imaging),
5. high input costs (tetrofosmin) compared to the alternative (MIBI), even in the favorable settings of a nuclear cardiology centre with a high-volume recruitment rate,
6. safety issues raised by the Croatian Agency for Medicinal Products and Medical Devices (monoclonal antibodies) [18],
7. regulatory and authorization issues between state agencies and local importers representing the manufacturer of a respective drug in 2015 (^123^I ioflupane),
8. fragmentation of competitive public bidding, resulting in inconsistent purchasing and pricing practices from one contracting authority to another,
9. unfavorable CHIF’s reimbursement policy (monoclonal antibodies, ^99m^Tc ECD, ^123^I ioflupane) and
10. altered pediatric urinary tract infection imaging guidelines (planar ^99m^Tc DMSA renal cortical imaging) [19–22].

In addition, a strong incentive to improve reimbursement positions, exerted by a major change in the hospital payment system and CHIF’s coding rules in early 2015 [23] has probably accelerated at least some of the former trends. In any case, ^201^Tl has been superseded by ^99m^Tc perfusion agents, whereas MUGA cardiac blood pool scans and ^99m^Tc besilesomab have been abandoned. Similarly, ^67^Ga and ^111^In imaging shows decreasing trend.

Increase in number of ^18^F FDG PET/CT procedures from 2010 to 2015 (Table 1) was because of installation of first medical cyclotron (2010), and later, PET/CT in a public hospital (2012), thus making this procedure more available. The reimbursement and the expansion of clinical indications covered by CHIF (2011) further fuelled the trend. More than half the European countries reported that the use of PET-CT for oncological imaging has increased and it is considered to be good practice in this application [6], provided that procedure standards, local regulations and evidence-based appropriateness criteria are well-observed. Nevertheless, the increase in the number of procedures (Fig 1) and decrease of average administered activity of 18F FDG in 2015 (Table 1) led to the similar contribution to the collective effective dose in both years (Fig 2). Similarly, an increase in the number of myocardial perfusion SPECT continues, given the high burden of coronary heart disease (CHD), high age-standardized mortality rate and an increase in 10-yr death rate from CHD in Croatia [24].

Differences in number of procedures and average administered activities found between 2010 and 2015 are results of technological development, better availability of NM services or radiopharmaceuticals etc. These differences show need of performing study like this periodically to identify possible trends, but also to raise awareness about the potential dose optimization and give guidance on how to reduce the dose, e.g. to optimize protection of patients in the most cost-effective manner.

## Supporting information

S1 File Data_NM_CRO_2010_15. Supporting information for Table 1 and Figs 1–4.
(XLS)

S2 File List and coding of NM procedures available in Croatia. (DOC)
