# Correction: Estimated collective effective dose to the population from nuclear medicine diagnostic procedures in Croatia: A comparison of 2010 and 2015

**DOI:** 10.1371/journal.pone.0183010

**Published:** 2017-08-03

**Authors:** The PLOS ONE Staff

The seventh author’s name is incorrect. The correct name is: Neva Girotto. The publisher apologizes for the error.
