# Supplementary material for: Estimated collective effective dose to the population from nuclear medicine diagnostic procedures in Croatia: A comparison of 2010 and 2015
Source: PLoS One. 2017 Jun 29;12(6):e0180057. doi: 10.1371/journal.pone.0180057 (PMC5491126; doi:10.1371/journal.pone.0180057)
Supplement: S2 File — (DOC) [file pone.0180057.s002.doc]

| **Studies** | **Procedures** | **Radiofarmaceutical** | **ACHI code** |  |
| --- | --- | --- | --- | --- |
| **Heart** | Myocardial perfusion (SPECT/gSPECT, rest, stress) | Tc-99m tetrofosmin, Tc-99m sestamibi | 61303-00, 61303-01,61307-00 | 2004 |
|  | Myocardial viability | Tl-201 | 90910-00 | 2014 |
|  | First pass angiocardiography | Tc-99m pertechnetate | 61320-00 | 2005 |
|  | Myocardial inervation | I-123 MIBG | 90910-00 | 2014 |
|  | Gated equilibrium radionuclide ventriculography | Tc-99m labeled RBC | 61313-00 | 2002 |
| **Bone** | Whole body (WB+local), three-phase scans | Tc-99m phosphonates | 61421-00, 61446-01 | 2009, 2010, 2011 |
|  | Bone marrow | Tc-99m labeled monoclonal antibodies, colloids | 61445-00, 61441-00 | 2009, 2011 |
| Brain | Transporter imaging (DAT) | I-123 ioflupane | 61402-00 |  |
|  | Perfusion | Tc-99m HMPAO | 61402-00 | 2000 |
|  | Brain death | Tc-99m DTPA | 61405-00 |  |
| **Lung** | Perfusion | Tc-99m MAA | 61328-00 ,613438-00 | 2006 |
|  | Ventilation | Tc-99m DTPA, Tc-99m technegas | 61340-00, 613438-00 | 2006 |
| **Kidney** | Renography, Diuretic renography, Captopril studies | Tc-99m MAG3, Tc-99m DTPA, I-131 OIH | 61386-00, 61390-00 | 2008 |
| **(genitourinary)** | Cortical scan | Tc-99m DMSA | 61386-01 | 2008 |
|  | Renography | Tc-99m DTPA | 61386-00, 61393-00 | 2008 |
|  | Direct/indirect radionuclide cystography | Tc-99m pertechnetate, Tc-99m MAG3 (DTPA) | 61397-00 | 2014 |
| **Thyroid** | Scan | Tc-99m pertechnetate | 61473-00 | 2014 |
|  | I-131 (whole body, diagnostic-oncology) | I-131 | 61426-00 | 2012 |
|  | Uptake and scan | I-131 | 61473-00 | 2014 |
| Gastrointestinal | Bleeding | Tc-99m labeled RBC, colloids | 61364-00 | 2007 |
|  | Liver hemangioma | Tc-99m labeled RBC | 61356-00 | 2014 |
|  | Liver + spleen (RES) | Tc-99m colloids | 61352-00 | 2014 |
|  | Spleen | TC-99m heat-damaged RBC | 61356-00 | 2014 |
|  | Hepatic (SPECT, oncology) | In-111 octreotide | 61396-00 | 2014 |
|  | Hepatobiliary scintigraphy | Tc-99m IDA derivatives | 61360-00, 61360-01 | 2014 |
|  | Meckel's diverticulum | Tc-99m pertechnetate | 61368-00 | 2007 |
| Infection  Inflammation | Labeled monoclonal antibodies  (planar+SPECT, WB + local) | Tc-99m NCA-95 Ab | 61433-00, 61434-00  61454-00, 61457-00 | 2012, 2013 |
|  | Ga-67 (WB, local) | Ga-67 citrate | 61429-00, 61454-00 | 2012, 2013 |
|  | Labeled leukocytes (planar+SPECT) | In-111 oxine, Tc-99m HMPAO | 61445-00, 61441-00 | 2009, 2011 |
| Endocrine | Adrenal (medulla) | I-123 MIBG | 61484-00, 61485-00 | 2014 |
|  | Adrenal | I-131 MIBG | 61484-00 | 2014 |
|  | Adrenal cortical scans | I-131 NP-59 | 61484-00 | 2014 |
|  | NET | Tc-99m octreotide, In-111 octreotide | 61396-00 |  |
|  | Parathyroid | Tc-99m sestamibi, Tl-201, pertechnetate | 61480-00 | 2014 |
| Salivary gland | Pertechnetate | Tc-99m pertechnetate | 61372-00 | 2001 |
| Lacrimal ducts | Pertechnetate | Tc-99m pertechnetate | 61495-00 | 2001 |
| Lymphoscintigraphy | Sentinel node + peripheral | Tc-99m nanocolloid | 61469-00 | 2005 |
| **Oncology (excl. thyroid, bone, NET)** | PET/CT | F-18 FDG, F-18 choline | 90905-02 | 2012 |
| Miscellaneous | RBC mass, plasma, blood volume | Cr-51 chromate, Tc-99m HSA | 12500-00 | 1863 |
|  | Schilling's test | Co-57, Co-58 | 21515-00 | 1863 |
|  | CSF flow | In-11 DTPA | 61409-00 | 2014 |
|  | Renal clearance (GF, blood sampling) | Cr-51 EDTA, Tc-99m DTPA | 12524-00 | 1863 |
|  |  |  |  |  |
|  |  |  |  |  |
| **Studies** | **Procedures** | **Radiofarmaceutical** | **ACHI code** |  |
| Radionuclide therapy |  |  |  |  |
| Thyroid | I-131 therapy |  | 16009-00 | 1795 |
|  | Benign (hyperthyroidism) | I-131 |  |  |
|  | Malignant (thyroid remnant ablation, metastases) | I-131 |  |  |
| Hematologic | Refractory B-cell NHL | Y-90 ibritumomab tiuxetan | 16003-00 | 1795 |
| Bone metastases | Palliation of pain | Sm-153 lexidronam | 16018-00 | 1795 |
| Neuroendocrine | MTC, pheochromocytoma | I-131 MIBG | 16009-00 | 1795 |
